# Toll Receptors Type-2 and CR3 Expression of Canine Monocytes and Its Correlation with Immunohistochemistry and Xenodiagnosis in Visceral Leishmaniasis

**DOI:** 10.1371/journal.pone.0027679

**Published:** 2011-11-30

**Authors:** Izabela Ferreira Gontijo de Amorim, Sydnei Magno da Silva, Maria Marta Figueiredo, Eliane Perlatto Moura, Rodrigo Soares de Castro, Tatjana Keesen de Souza Lima, Nelder de Figueiredo Gontijo, Marilene Suzan Marques Michalick, Kenneth John Gollob, Wagner Luiz Tafuri

**Affiliations:** 1 Departamento de Patologia Geral, Escola de Medicina, Universidade Federal de Minas Gerais, Minas Gerais, Brasil; 2 Departamento de Parasitologia, Instituto de Ciências Biológicas, Universidade Federal de Minas Gerais, Minas Gerais, Brasil; 3 Departamento de Parasitologia, Escola de Medicina, Universidade Federal de Minas Gerais, Minas Gerais, Brasil; 4 Departamento de Bioquímica e Imunologia, Instituto de Ciências Biológicas, Universidade Federal de Minas Gerais, Minas Gerais, Brasil; Universidad Nacional, Costa Rica

## Abstract

The aim of the present study was to investigate TLR2 expression in peripheral blood monocytes from dogs naturally infected with *Leishmania (Leishmania) infantum* to determine whether it correlates with CD11b/CD18 (CR3) expression, and to evaluate the potential of dogs as sources of infection using phlebotomine xenodiagnosis. Forty eight dogs were serologically diagnosed with *L. infantum* infection by indirect immunofluorescence antibody test (IFAT) and enzyme linked immunosorbent assay (ELISA). Parasitological exams from bone-marrow aspirates were positive by PCR analysis. All dogs were clinical defined as symptomatic. Ear skin tissue samples were obtained for immunohistochemistry (IHQ) analysis. The potential of these dogs as a source of infection using phlebotomine xenodiagnosis (XENO) was evaluated. Flow cytometry was carried out on peripheral blood mononuclear cells using superficial receptors including CD14, CD11b, TLR2 and MHCII. IHQ ear skin tissue parasite load and XENO where done where we found a strict correlation (r = 0.5373). Dogs with higher expression of MFI of CD11b inside CD14 monocytes were represented by dogs without parasite ear tissue load that were unable to infect phlebotomines (IHQ^−^/XENO^−^). Dogs with lower expression of MFI of CD11b inside CD14 monocytes were represented by dogs with parasite ear tissue load and able to infect phlebotomines (IHQ^+^/XENO^+^) (p = 0,0032). Comparable results were obtained for MFI of MHCII (p = 0.0054). In addition, considering the population frequency of CD11b^+^TLR2^+^ and CD11b^+^MHCII^+^, higher values were obtained from dogs with IHQ^−^/XENO^−^ than dogs with IHQ^+^/XENO^+^ (p = 0.01; p = 0.0048, respectively). These data, together with the TLR2 and NO assays results (CD11b^+^TLR2^+^ and NO with higher values for dogs with IHQ^−^/XENO^−^ than dogs with IHQ^+^/XENO^+^), led to the conclusion that IHQ^−^/XENO^−^ dogs are more resistant or could modulate the cellular immune response essential for *Leishmania* tissue clearance.

## Introduction

Toll-like receptors (TLRs) function as pathogen recognition receptors (PRRs) that recruit active signaling molecules involved in innate immunity [1]. These receptors located on the plasma membrane or internal membranes of macrophages, dendritic cells (DCs), NK cells and T and B lymphocytes, recognize “pathogen-associated molecular patterns” (PAMPs) such as glycolipids, peptidoglycans and lipopeptides, which are produced only by microorganisms and not by host cells [2]. After this recognition there is a spectrum of regulatory inflammatory cytokines production by the host. Evidences that TLRs are important microbial sensors came from models of infection in TLR-deficient mice. In addition, reported polymorphisms in certain TLRs and signaling adaptors predict susceptibility to infectious diseases [3]. A total of 11 human and 13 mouse TLRs have been identified and each responds to distinct PAMPs, leading to the activation of specific signaling pathways. Among TLRs located on internal membranes, TLR3, TLR7, TLR8 and TLR9 have been described and TLR1, TLR2, TLR4, TLR5 and TLR6 are located on plasma membranes [4].

The majority of work concerning TLRs has involved bacterial and fungal pathogens, but some studies have suggested that they may play a role in recognizing protozoan parasites beyond glycosylphosphatidylinositol (GPI) anchors and glycoinositol phospholipids (GIPL) [5]. *Leishmania* cell surfaces are dominated by GPI-anchored and GPI-related molecules and abundant GIPLs that are not attached to a protein and tend to form dense layers on the parasite surface, above other GPI-anchored molecules such as lipophosphoglycan (LPG) [6]. It has been demonstrated that LPG in *L. major* activates cells of the innate immune response through TLR-2. Therefore, owing to its structural characteristics and its GPI anchor, LPG represents a *Leishmania* ligand for TLRs [7], [8]. The study carried out by Hawn et al. (2002) [9] was the first to evaluate TLRs in *Leishmania* infection. The authors demonstrated that there was less IL-1alfa mRNA expression in MyD88 (intracellular signaling protein) knockout mice. However, this study evaluated *in vitro* cytokine promoter (adaptor protein MyD88) and not TLR activation. Following this work, de Veer et al. 2003 and Beker et al. (2003) [7], [8] demonstrated that LPG was associated with an increase in TNF-α levels after NF-kB activation by TLR2, suggesting that three molecules of LPG could aggregate with one molecule of TLR2. Therefore, these studies appear to invalidate the concept of the unresponsiveness of TLR2 in leishmaniasis [10].

Several non-TLR receptor chains including CD14, MHC class II and the integrin-like CD11b/CD18 receptors (CR3 - complement receptor type 3) cooperate with TLRs in recognizing PAMPs [11]. Therefore, the aim of this study was to investigate the expression of TLR2 in peripheral blood monocytes cells of symptomatic dogs naturally infected with *Leishmania (L.) infantum (syn*: *L. chagasi)* [12] and its correlation with CR3 expression. Moreover, skin parasite tissue load assessed by immunohistochemistry associated with the potential of these symptomatic dogs as a source of infection using phlebotomine xenodiagnosis were evaluated, applying this technique to detect and isolate the pathogen using the natural arthropod vector [13]. Although it cannot be considered an ultimate routine technique in diagnosing *Leishmania*, it has significant epidemiological implications [14].

## Materials and Methods

### Animals

The study was submitted to and approved by the CETEA/UFMG (Comitê de Ética em Experimentação Animal/Universidade Federal de Minas Gerais), protocol 211/2007 (valid to March 12, 2013). All procedures involving animals were conducted according to the guidelines of the Colégio Brasileiro de Experimentação Animal (COBEA).

Forty-eight (48) mongrel dogs of unknown age (adult dogs) and both genders were obtained from the Control Zoonosis Center of the Municipality of Ribeirão das Neves, Belo Horizonte Metropolitan area, Minas Gerais (MG) state, Brazil. Dogs were diagnosed with *Leishmania (Leishmania) infantum* infection using indirect immunofluorescence antibody test (IFAT) (Title >1∶40 dilution) and enzyme linked immunosorbent assay (ELISA) (optical density >100; 1∶ 400 dilutions), and assigned for euthanasia. Previous work using other dogs obtained from the metropolitan area of Belo Horizonte demonstrated the presence of *L. infantum* using polymerase chain reaction (PCR). Indeed, liver tissue from a naturally infected dog presented with a conserved region of kinetoplastidae (kinetoplast mini-circle DNA or kDNA) and hybridization with kDNA probes verified the presence of *L. infantum* [15].

Prior to inclusion in this study, dogs received anti-helmintic and anti-ectoparasitic treatment and were immunized against parvovirus, distemper, leptospirosis, parainfluenza and hepatitis (HTLP 5CV-L vaccine Pfizer®). They were maintained in kennels at the Department of Parasitology of Instituto de Ciências Biológicas (ICB), Universidade Federal de Minas Gerais (UFMG), Belo Horizonte, MG, Brasil. Commercial dog food and water were provided *ad libitum*. Five non infected dogs (NID) with negative serological exams (IFAT and ELISA) for *Leishmania* composed the control group.

### Clinical examination

Physical clinical examinations were carried out on forty eight symptomatic dogs and the data transferred to individual assessment forms. This procedure was divided into six items: (1) body condition; (2) level of attentiveness; (3) vital signs; (4) palpate peripheral lymph nodes (sub-mandibular, cervical and popliteo); (5) dermatological signs and (6) other systems evaluation. Animals were categorized by the presence of clinical signs of LVC.

After clinical examination, animals were classified as symptomatic dogs with at least one clinical alteration such as a dermatological change (alopecia, dry exfoliative dermatites or ulcers), ocular and conjunctival disorders, onychogryphosis, musculoskeletal disorders, lymphadenophathy and weight loss [16]. Hematological and biochemical examinations of peripheral blood were carried out in addition to physical clinical examination, but it was not exclusively used to define the clinical status of naturally infected animals.

### Blood sample collection

Twenty five milliliter of peripheral blood samples were collected from each naturally infected dogs (n = 48) by jugular venipuncture after trichotomy and local antisepsis, in 10cc disposable sterile syringes with 21G1 needle (0.80 mm x 25 mm). After, 10 ml of blood were transferred to tubes containing ethylenediamine tetraacetic acid (K_3_EDTA) for the flow cytometry, being that, the plasma sample were collected and stored at −20°C for further nitric oxide (NO) assay. Another blood sample (5.0 ml) was send to Laboratory Diagnostics TECSA Pet (Belo Horizonte, MG, Brazil) for complete erythrogram and total count of platelets (ABCvet®, ABX, France). Also, samples without anticoagulant was obtained for biochemical tests carried out by conventional spectrophotometria method (Celma®, Brazil) for the measurement of urea, creatinine and serum proteins. Blood samples of five non infected dogs (NID) were collected to flow cytometry and NO procedures.

Other blood samples without anticoagulant of infected and non infected dogs were obtained and send to serological laboratory of Departament of Parasitology, ICB/UFMG, Brazil, for serological tests as IFAT and ELISA, described as follow.

### Serological procedures

#### Indirect immunofluorescence antibody test (IFAT)

IFAT was used to detect anti-*Leishmania* antibodies according to a modified version of the method of Rosario et al. (2005) [17]. The antigen was prepared from *L. infantum* MHOM/BR/1967/BH46 promastigotes and fixed in slides. Serum samples were previously diluted at 1∶40 in phosphate buffered saline (PBS) and 25 µL placed on the demarcated regions of the slide. The slide was incubated in a humid chamber at 37°C for 30 min, washed with PBS, and dried at room temperature. Then 25 µL of a commercially available fluorescein-conjugated anti-dog IgG (Bethyl® Laboratories, Montgomery, TX, USA) diluted at 1∶1500 in PBS containing 2% of Tween (Tween® 80, Merck, Germany) were added in each demarcated region of the slide, followed by new incubation, washing and drying. Slides were examined under an Olympus BX 41® fluorescent microscopy (Tokyo, Japan) and samples presenting fluorescence at the dilution of 1∶40 were considered to be positive. Positive controls consisted of serum obtained from infected dogs, whilst serum from non-infected animals was employed as negative control.

#### Enzyme linked immunosorbent assay (ELISA)

Determinations of anti-*Leishmania* IgG were carried out using the ELISA technique (Voller et al., 1979) [18] with modifications (de Amorim et al., 2010) [16]. *Leishmania* soluble antigen (LSA) was derived from *L. chagasi* strain MHOM/BR/1967/BH46 promastigote forms ruptured ultrasonically. Aliquots (100 µL) of soluble antigen dissolved in 0.05 M carbonate buffer (pH9.6) to a final concentration of 2 µg/mL were transferred to individual wells of a 96-well microplate and incubated overnight at 4°C. The coated wells were washed five times with PBS containing 0.2% Tween-20, and the antigenic sites were saturated with 150 µL of PBS containing 0.2% Tween-20 and 2% casein (Sigma®^,^ St Louis, MO, USA; product # C0376) for 30 min at 37°C, washed three times with PBS, and a 100 µL aliquot of serum sample (diluted 1∶400) was placed into each well. Plates were incubated for 45 min at 37°C, washed five times with PBS, and a 100 µL aliquot of diluted enzyme-labelled immunoglobulin was added to each well. The titre of rabbit-anti-canine-IgG conjugates (Sigma®; product # A6792) was 1/10.000. Following incubation for 45 min at 37°C plates were washed five times with PBS, and a 100 µL aliquot of 4% (w/v) ortho-phenylenediamine in phosphate/citrate buffer (pH 5) containing 4 µL of 30 (v/v) hydrogen peroxide was added to each well. The reaction mixture was incubated at 37°C for 10 min. Reaction was stopped by addition of 25 µL of 2 M sulphuric acid to the well, and absorbance was measured at 492 nm using a BioRad (São Paulo, SP, Brazil) model 550 ELISA reader. The cut-off point was the mean absorbance reading of the VL-negative controls plus two-times the standard deviation.

### Parasitological Analysis

#### PCR

Specific PCR carried out on bone marrow aspirates was used to confirm that animals were infected with *L. infantum*. Bone marrow aspirate (1.0 ml) from the iliac crest was collected and stored at −80°C until required [19]. In parallel, healthy ear skin tissue samples were obtained using a 5 mm punch and these tissue samples were immediately fixed in 10% formalin for immunohistochemistry (IHQ) analysis [15]. For these distinct procedures, dogs were previously subjected to general anesthesia using a combination of 1.0 mg/Kg xylazine chlorhydrate (2%) (Anesedan®, Vetbrands, Brasil) and 10 mg/Kg ketamine chlorhydrate (10%) (Agener®, União Química Farmacêutica S/A, São Paulo, SP, Brasil) using intramuscular injection.

For PCR, 50 µL bone marrow aspirates were extracted using a "DNeasy® Blood & Tissue Kit" (Qiagen Inc., Valencia, CA, USA) according to the manufacturer's instructions. The oligonucleotide primers LV_1_ (5′ ACGAGGTCAGCTCCACTCC 3′) and LV_2_ (5′ CTGCAACGCCTGTGTCTACG 3′) used for DNA amplification were specific for a repetitive DNA sequence in *L. infantum* [20]. The PCR reaction and amplification were carried out according to da Silva et al. (2009) [21]. Amplified PCR products were analyzed on 5% silver-stained polyacrylamide gels.

*RT-PCR*

The ear skin parasite load was determined by real time PCR (RT-PCR) according to Alves et al. (2009) [22]. Total DNA extraction from skin samples was carried out with the aid of DNeasy Blood and Tissue Kit® (Qiagen, USA) following the manufacturer's instructions. In order to quantify parasite burdens, primers described by Bretagne et al. (2001) [23] that amplified a 90 bp fragment of a single-copy-number gene of DNA polymerase of *L. chagasi* (GenBank accession number AF009147) were used. PCR was carried out in a final volume of 25 µl containing 200 nM forward and reverse primers, 1× SYBER GREEN reaction master mix (Applied Biosystems, USA) and 5 µl of template DNA. PCR conditions were as follows: an initial denaturation step at 95°C for 10 min followed by 40 cycles of denaturation at 95°C for 15 s and annealing/extension at 60°C for 1 min. Standard curves were prepared for each run using known quantities of TOPO PCR 2.1 plasmids (Invitrogen, USA) containing genes of *L. chagasi*. Same procedure was carried out for β-actin gene (307 bp fragment) in order to verify the integrity of the samples and to normalize the initial concentrations of DNA [22]. The number of copies of *L. chagasi* in the samples was adjusted using the β-actin correction factor obtained for each sample. Reactions were processed and analyzed in an ABI Prism 7500 -Sequence Detection System (Applied Biosystems, EUA).

#### Immunohistochemistry

Deparaffined slides were hydrated and incubated with 4% hydrogen peroxide (30vv) in 0.01 M Phosphate Buffered Saline (PBS; pH 7.2) to block endogenous peroxidase activity, followed by incubation with normal goat serum (1∶100 dilution) to block non-specific immunoglobulin absorption. Heterologous hyperimmune serum from dogs naturally infected with *L. infantum* (IFAT titer ≥1∶40) was diluted 1∶100 with 0.01 M PBS and employed as the primary antibody. Slides were incubated in a humid chamber at 4°C for 18-22 h, washed with PBS, incubated with biotinylated goat anti-mouse and anti-rabbit Ig (Dako, Carpinteria, CA, 192 USA; LSAB2 kit), washed in PBS, and incubated with streptavidin-peroxidase complex (Dako; LSAB2 kit) for 20 min at room temperature. Slides were treated with 0.024% diaminobenzidine (Sigma) and 0.16% hydrogen peroxidase (30vv), dehydrated, cleared, counterstained with Harris*'*s hematoxylin and mounted with cover slips. This immunohistochemistry method was carried out using a secondary antibody not specific to canine immunoglobulin, characterizing a cross-immune reaction as an alternative method for detecting *Leishmania* amastigotes in paraffin-embedded canine tissues previously described by Tafuri et al. (2004) [24].

#### Xenodiagnosis

Four-day-old females of *Lutzomyia longipalpis* from the colony of the Laboratory of Physiology of Haematophagous Insects (Department of Parasitology, ICB/UFMG) were used to perform xenodiagnosis. The dogs were submitted to general anesthesia as described previously and then the internal surface of the right ear was shaved. Based on the protocol of da Silva et al. (2010) [19], with some modifications, 50 females of *L. longipalpis* were placed in a round plastic boxe named *FleboContainers*, and the sand flies were allowed to feed directly on the right ear of the infected dog for 40 min. After the blood meal, the sand flies were fed daily with a 50% fructose solution in distilled water and kept at 28°C, 60% humidity, in the Laboratory of Physiology of Haematophagous Insects insectary for five days. On the fifth day, the females of *L. longipalpis* were dissected in a drop of PBS solution and midguts were examined under an optical microscope at 400× magnification to verify the presence or absence of promastigote forms.

### Flow Cytometry

To isolate peripheral blood mononuclear cells (PBMC) of infected and non infected dogs (NID), the blood was gently overlaid on Ficoll-Hypaque (Histopaque® 1.077 – Sigma, USA) at a ratio of 1 Ficoll/2 blood, and centrifuged (300 x *g*, 40 min, 14°C). The PBMCs, collected at the Ficoll/plasma interface, were collected and transferred to another sterile tube. The volume was adjusted to 15 ml with PBS-W (0.15 M, 8 g/L of NaCl, 2 g/L of KCl, 2 g/L of KH_2_PO_4_ and 1.15 g/L of Na_2_HPO_4_, pH 7.2 with 0.5% bovine serum albumin, Inlab®, and 0.1% Na_3_N) and centrifuged at 300× *g* for 10 min at 4°C. The supernatant was discarded and 5 ml lysis solution (FACS lysing solution; Becton Dickinson, San Diego, CA, USA) was added to the pellet, followed by incubation for five min at room temperature. The volume was adjusted to 15 ml with PBS-W followed by further centrifugation and washing with PBS-W. The final volume was adjusted to 1 ml and the concentration was adjusted to 1×10^7^ cells/ml.

In 96-well U-bottom plates (Limbro Biomedicals®, Aurora, OH, USA), 20 µl of leukocyte suspension were incubated at 4°C for 30 min in the dark and in the presence of 20 µl anti-canine or anti-human cell surface marker monoclonal antibodies (mAbs) diluted to 10% in PBS. A range of mononuclear cell surface markers were used including diluted purified anti-canine CD11b 1∶50 (mouse IgG1, clone MCA 1777S, Serotec®, USA); anti-human CD14:RPE-Cy5 1∶50 (mouse IgG1, CLONE 61D3, AbD Serotec®, USA), anti-human TLR2:RPE 1∶20 (mouse IgG_2a_, clone 2B4A1, SouthernBiotech®, Canada and USA) and mouse anti-canine MHCII 1∶20 (mouse IgG1, MCA2037S, Serotec®, USA). Non-conjugated, purified antibodies were conjugated using a Zenon tricolor kit (Molecular Probes® – Z-25080) as described by the manufacturer. The cells were incubated with labeled antibody solutions for 20 min at 4°C. After staining, the preparations were washed with 0.1% Na_3_N in PBS, fixed with 200 µl 2% formaldehyde in PBS and kept at 4°C until data were acquired by flow cytometry (FACScan, Becton & Dickinson®, San Jose, CA, USA).

The cells were run on an analytical flow cytometer equipped with a laser emitting at 488 nm (FACSVantage, Becton-Dickinson®, San Diego, CA, USA), whole cells were distinguished from fragments by gating based on the forward and side scatter signals and a minimal of 50.000 events were acquired for each preparation [25].

**Figure 1:**
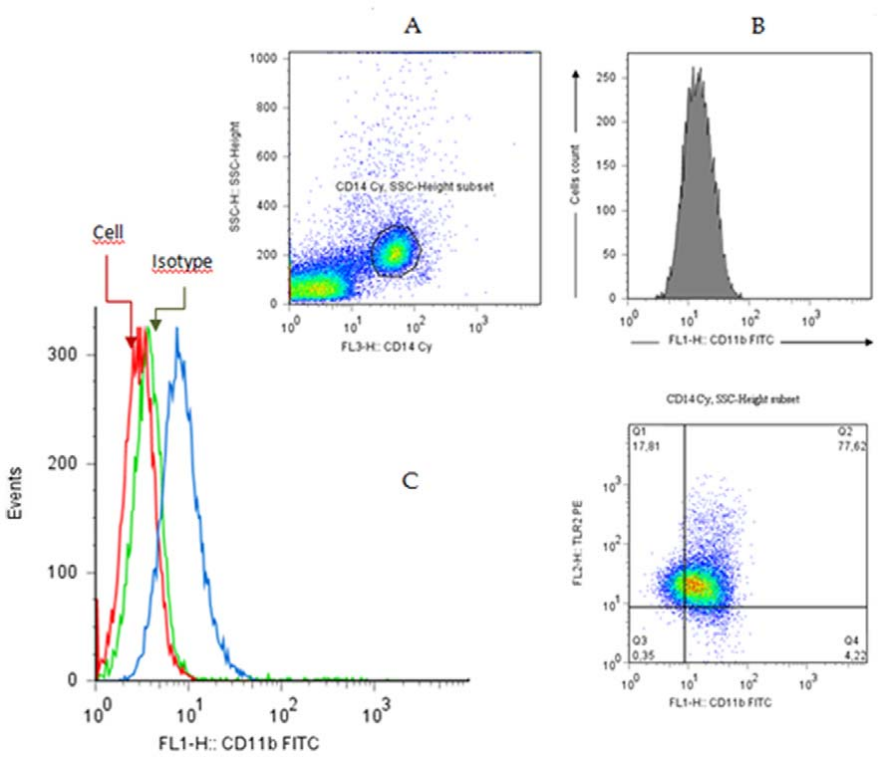
Identification of monocytes subpopulation of the peripheral blood mononuclear cells (PBMC) in naturally infected dogs with *Leishmania (L.) infantum.* Panel A has depicted the gate of monocytes based on SSC *versus* CD14/FL3 expression dot plot (SSC^intermediate^CD14^hight+^) subpopulation; Panel B, geometric mean fluorescent intensity (MFI) of CD11b *versus* cells number (previous subpopulation selection). Panel C (representative dotplot) showing gates that were set, based on negative controls (cell and isotype)

Cells were analyzed using the program FlowJo® (Tree Star. Inc., Ashland, OR, USA). To assess the monocyte population the flowing strategy using anti-CD14 RPE-Cy5 *versus* SSC dot plot distribution was adopted and it was identified as SSC^intermediate^CD14^high+^. The phenotypic aspects of the circulating monocytes were expressed in two different forms: percentage (%) of cells expressing a given phenotypic marker, using cell and isotype control cut-off, with a bimodal distribution, and geometric mean fluorescence intensity (MFI). The latter approach was used for semi-quantitative expression of phenotypic markers with a unimodal distribution, as the entire monocyte population expressed such phenotypic markers constitutively, and values were obtained on a logarithmic scale (Figure 1).

### Quantification of nitric oxide (NO) levels in dogs plasma

The concentration of nitrite (NO2^-^) in all infected and non infected (NID) dogs plasma aliquots was measured using the Griess reaction (1%sulphanylamide, 0.1% naphthylethylene-diamide-dihydrochloride and 2.5% phosphoric acid, Sigma®, St. Louis, MO, USA) [26]. Briefly, a 25 µl of plasma was mixed with 25 µl nitrate reductase (overnight/37°C). Then, 50 µl of Griess reagent was added to each well, except the nitrite standard solution. Following 10 min of incubation at room temperature, in the dark, the absorbance was measured at 540 nm, using a microplate reader. Each sample was assayed in duplicate and the concentration of nitrite was determined by interpolation from a standard curve constructed using sodium nitrite solutions of known concentration in the range 0-100 µM. To discount the interference of nitrites already present in the plasma samples, data was calculated taking into account the blank for each experiment. The results were first expressed as nitrite concentration (µM).

### Statistical analysis

Initially the parametric or non-parametric nature of the data was evaluated. The non-parametric aspects of the data were evaluated using a Mann-Whitney test and Kruskal-Wallis and Spearman's rank correlation coefficient. The parametric aspects of the data were evaluated using One Way ANOVA. These analyses were done using GraphPad Prism® 3.03 software package (San Diego, CA, USA). In all cases the statistical difference were considered significant when the probabilities of equality p-values were <0,05.

## Results

### Clinical Evaluation

**Figure 2:**
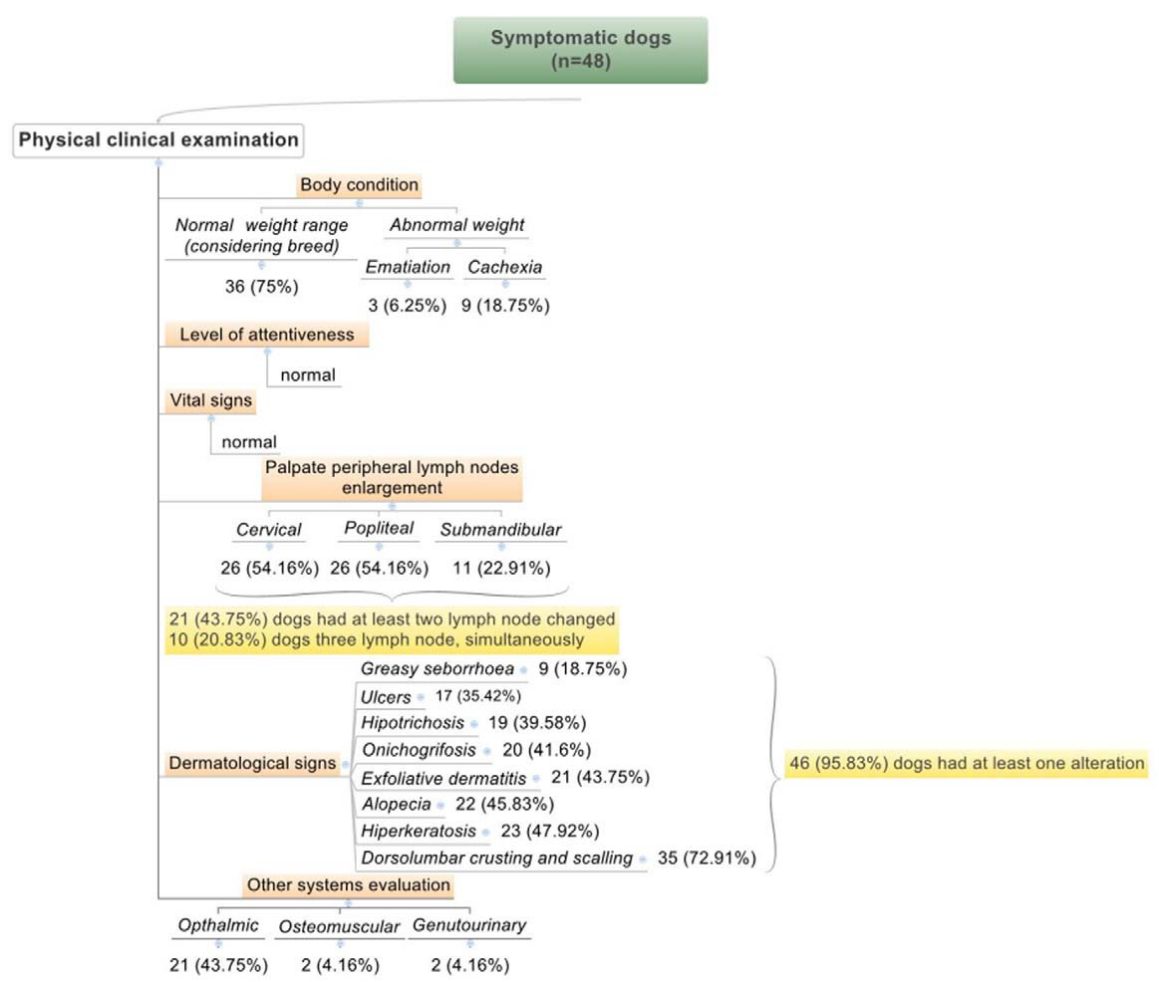
Clinical findings of forty-eight dogs naturally infected with *Leishmania (L.) infantum*, Belo Horizonte, Minas Gerais, Brazil.

After physical clinical analysis, thirty six dogs (75%) were within the normal weight range (considering breed), nine (18.75%) had abnormal weight classified with cachexia and three (6.25%) had emaciation. Cervical, popliteal and submandibular lymph node enlargement was present in 26 (54.16%), 26 (54.16%) and 11 (22.91%) dogs, respectively. Moreover, 21 (43.75%) dogs had at least two lymph nodes changed and ten (20.83%) had involvement of three lymph nodes simultaneously. Dermatological examination demonstrated that 46 (95.83%) dogs had at least one dermatological alteration and 32 (66.7%) had at least two alterations simultaneously. Other clinical remarks with clinical importance were the ophthalmic alterations in 21 dogs (43.75%) (Figure 2). The study of erythrocyte values in dogs naturally infected with *L. infantum* demonstrated that 16 (33.33%) of 48 animals had changes in the functional status of eritron, being that all of which were below the reference limits for the three parameters analyzed: circulating red blood cell (RBC), hematocrit and hemoglobin. When these parameters were evaluated by considering the mean corpuscular volume and hemoglobin concentration, anemia was classified as: normocytic normochromic in 14 of 16 dogs (87.5%) and normocytic hypochromic in two of 16 dogs (12.5%) analyzed. In addition, thrombocytopenia was observed in 6 of 16 dogs (37.5%). Eight other animals had thrombocytopenia without changes in eritron values. Therefore, thrombocytopenia was present in 14 of 48 dogs (29.2%). The creatinine levels were increased only two infected dogs (4.16%), but the urea values were increased in 42 dogs (85.42%). Serum liver enzymes alamine aminotransferase (ALT) and aspartate aminotransferase (AST), indicators of hepatocellular injury, had normal levels in all dogs analyzed, whilst, alkaline phosphatase (AF), an indicator of cholestasis, was increased in 11 of the 48 infected dogs (22.91%). Disproteinemia was observed in 25 of 48 infected dogs (52.1%), evidenced by hyperproteinemia and hypoproteinemia in 23 (47.91%) and two (4.12%) dogs, respectively. Furthermore, the albumin/globulin ratio was altered in 34 dogs (70.83%) when considering A/G ≤0.6, the prognostic critical value for disease development, and in five dogs (10.42%) when considering ≤0.7 A/G <0.8. Hypergammaglobulinemia was observed in 23 of 48 infected dogs (47.92%) and hypoalbuminemia in 17 (35.42%), and both parameters were simultaneously observed in 12 dogs (25.0%).

### Parasitological analyses

Evaluation of the PCR amplification of kDNA from bone marrow aspirates yielded positive results for all (n = 48) dogs analyzed.

**Figure 3:**
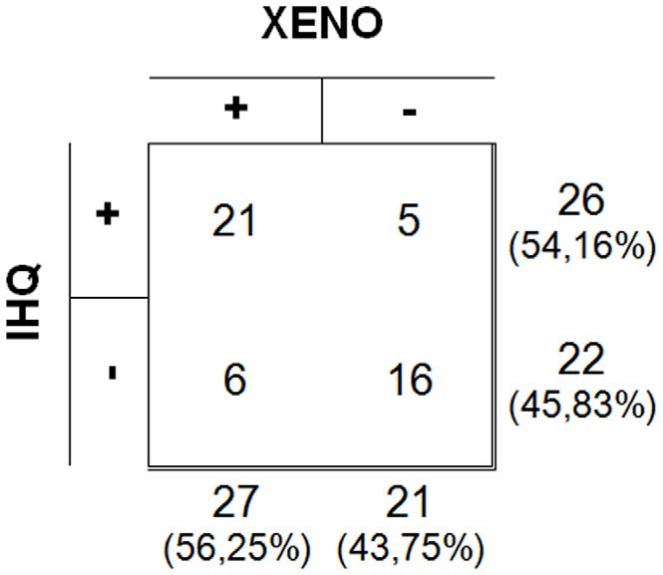
Comparison between the results of immunohistochemistry (IHQ) and xenodiagnosis (XENO) in forty-eight dogs naturally infected with *Leishmania (L.) infantum*, in Belo Horizonte, Minas Gerais, Brazil.

Immunohistochemistry (IHQ) of ear skin and xenodiagnosis (XENO), achieved positivity in 26 (54.16%) and 27 (56.25%) cases, respectively (Figure 3).The correlation was positive between these two parameters (r =  0.5373; p = 0.001).

**Figure 4:**
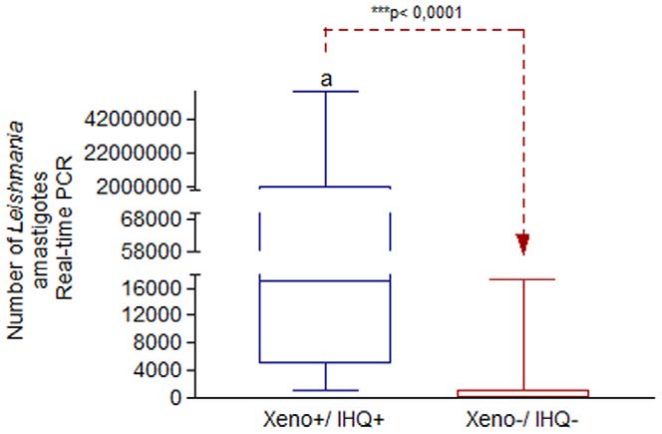
Quantitative study by Real Time PCR (RT-PCR) of the skin tissue parasite load. Dogs naturally infected with *Leishmania (L.) chagasi* of double positive XENO^+^/IHQ^+^ (n = 17) *versus* double negative XENO-/IHQ- (n = 16) groups were analyzed. The load was carried out using RT-PCR with primers specific for a simple-copy gene of DNA polymerase of *Leishmania chagasi.* Xeno^+^/IHQ^+^ (mean =  5.440.292,60), Xeno^−^/IHQ^−^ (mean =  1.495,217). Significant differences, at a level of 5% of probability, between cohorts were identified by the lower case letter (a) through Mann-Whitney Test.

The presence and quantification of the *Leishmania* skin tissue parasite burden of all naturally infected dogs were assessed by RT-PCR. These results showed double negative IHQ^−^/XENO^−^ group with a statistical significant lower mean (mean = 1.495,22; p = 0.0001) of parasite burden when compared with those of double positive ones (Figure 4).

### Flow Cytometry

CD14 has been demonstrated to be involved in the co-recognition of various TLR ligands by TLR2 and CD11b/CD18 receptors (CR3) (non-TLR receptor) cooperate with TLRs. Therefore, any correlation between CD11b and TLR2 inside SSC x CD14 of a canine monocyte positive population was investigated. A strong positive correlation with a Spearman r = 0.6937, p<0.001, was obtained.

CD11b and TLR2 semi quantitative analyses were performed in peripheral blood monocytes cells and the results were expressed as the geometric mean fluorescence intensity (MFI). A comparison inside the immunohistochemistry (IHQ) and xenodiagnosis (XENO) groups was carried out, considering the positive and negative results. The MFI of CD11b was statistical different between negative (mean = 17.296) and positive cases (mean = 13.369) of the IHQ group, (p = 0.0390). Likewise, differences between negative (mean = 20.398) and positive cases (mean = 11.217) of the XENO groups were calculated (p = 0.0004). Considering the MFI of TLR2, the average values for negative IHQ were higher than values obtained for the positive IHQ group, but this was not significant. On other side, there was significant difference between negative (mean = 40.188) and positive (mean = 17.278) XENO groups, (p = 0.0038). The values obtained for the CD11b^+^TLR2^+^ monocytes frequency were statistically different for both groups; negative IHQ (mean = 0.4717) and negative XENO (mean = 0.4511) groups had statistically higher mean values than the respective positive-ones, (p = 0.002).

**Figure 5:**
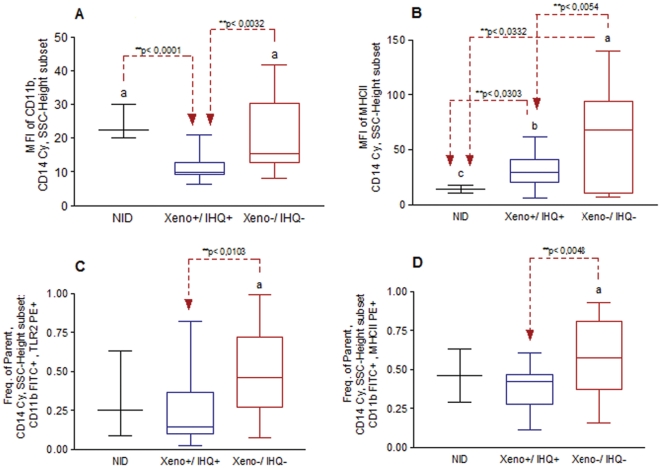
Flow cytometry analysis considering distinct phenotypical kinetics of monocytes (SSC^intermediate^CD14^hight+^) taking into account the non-infected dogs (NID), immunohistochemistry (IHQ) and xenodiagnosis (XENO) results. Panel A and B represent geometric mean fluorescent intensity (MFI) of CD11b and MHC II, inside previously select monocyte subpopulation, taking into consideration NID (n = 5) *versus* double positive XENO^+^/IHQ^+^ (n = 21) *versus* double negative XENO^−^/IHQ^−^ (n = 16) results. Panel C and D represent the percentage of fluorescent cells within the population CD11bFITC^+^.TLR2PE^+^ and CD11bFITC^+^.MHCIIPE^+^ considering NID (n = 5) *versus* double positive IHQ^+^/XENO^+^ (n = 21) *versus* double negative IHQ^−^/XENO^−^ (n = 16) results. MFI and Parent frequencies of constituent groups: CD11b - NID (mean =  24.016, 50^th^ percentile = 22.350), Xeno^+^/IHQ^+^ (mean = 10.83, 50^th^ percentile = 9.700), Xeno^−^/IHQ^−^ (mean = 19.23, 50^th^ percentile = 15.320); MHC class II - NID (mean = 14.32, 50^th^ percentile = 14.04), Xeno^+^/IHQ^+^ (mean = 30.35, 50^th^ percentile = 29.03), Xeno^−^/IHQ^−^ (mean = 61.64, 50^th^ percentile = 68.23); CD11b^+^.TLR2^+^ - NID (mean = 0.3276, 50^th^ percentile = 0.2500), Xeno^+^/IHQ^+^ (mean = 0.2687, 50^th^ percentile = 0.1410), Xeno^−^/IHQ^−^ (mean = 0.4806, 50^th^ percentile = 0.4620); CD11b^+^.MHCII^+^ - NID (mean = 0.4492, 50^th^ percentile = 0.4600), Xeno^+^/IHQ^+^ (mean = 0.3851, 50^th^ percentile = 0.4200), Xeno^−^/IHQ^−^ (mean = 0.5765, 50^th^ percentile = 0.5755). Significant differences, at a level of 5% of probability (p<0.05), between cohorts are identified by the lower case letter (a, b and c) through Kruskal-Wallis and One-way ANOVA

To confirm and extend the findings, a correlation of the same parameter, described above, was performed, taking into consideration the double positive and double negative IHQ/XENO groups in comparison to non infected dogs (NID). These results showed that MFI of CD11b was higher in non-infected dogs (NID) (mean = 24.016; p = 0.0001) and in a double negative IHQ^−^/XENO^−^ group (mean = 19.237; p = 0.0032) than those group characterized by double positive ones (IHQ^+^/XENO^+^). The MFI of MHCII was statistically different among all evaluated groups. The NID group achieved statistical lower values (mean = 14.32, p = 0.0303 and p = 0.0332) when compared to double positive (mean = 30.35) and negative (mean = 61.64) XENO/IHQ groups, respectively. A similar pattern was showed by XENO^−^/IHQ^−^ group were statistically higher values was found in comparison to the positive ones. Likewise CD11b^+^TLR2^+^ (mean = 0.4806; p = 0.01) and CD11b^+^MHCII^+^ (mean = 0.5765; p = 0.0048) parent frequencies were statistically higher in dogs in which it was not possible to locate amastigotes forms of *Leishmania* in ear skin tissue or even capacity to infect *L. longipalpis* when compared with the IHQ^+^/XENO^+^ group. In the other side, NID group did not show any results with significant differences when compared to the cohorts (Figure 5).

### Nitric Oxide (NO) Analysis

**Figure 6:**
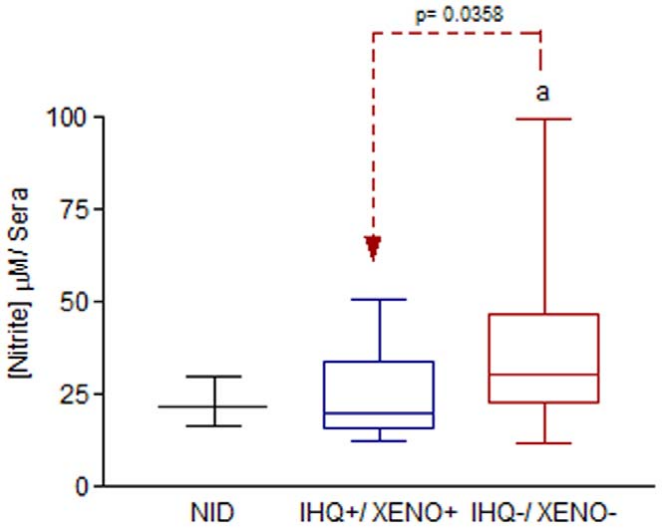
Nitric Oxide (NO) plasma levels of non-infected dogs (NID) and naturally infected dogs with *Leishmania (L.) infantum*, Belo Horizonte, Minas Gerais, Brazil. As an indirect measurement of NO production, the Griess reaction was used to determine the nitrite levels. These results are expressed in micromolar (µM). A comparison inside groups NID (mean = 21.93 µM) and dogs IHQ and XENO double positive (IHQ^+^/XENO^+^) (mean = 24.11 µM) or negative IHQ^−^/XENO^−^ (mean = 39.19 µM) was carried out. Significant differences, at a level of 5% of probability (p<0.05), between cohorts are identified by the lower case letter (a) through Kruskal-Wallis Test (p = 0.0358)

A comparison of NO levels was done inside the IHQ, XENO and NID groups. We found that IHQ^−^/XENO^−^ (mean = 39.19, p = 0.0358) dogs showed higher values than IHQ^+^/XENO^+^ dogs (mean = 24.11). In the other side, NID group did not show any results with significant differences when compared to the cohorts (Figure 6).

## Discussion

After physical clinical analysis of symptomatic dogs, in according to the literature concerning canine visceral leishmaniasis (CVL), the principal alterations identified were dermatological lesions (95.83%) [27], [28], [29]; [30]. In relation to the haematological and biochemistry clinical studies, an impaired eritron status was observed associate to normocytic/normochromic anemia in 14 of 48 of infected dogs (29.16%). Also, a albumin/globulin inversion ratio (A/G ≤0.6) was observed in 34 of 48 infected dogs (70.83%) and disproteinemia was detected in 25 of 48 infected dogs (52.1%) [31]. This is a prognostic critical value for disease development [27], [28], [32]. Other alterations observed were higher serum values for alkaline phosphatase (AF) in 11 dogs (21.15%). However, in this study modest laboratory signs of hepatic damage (ALT and AST) can not satisfactorily explain an increase in AF, considering that all infected dogs had normal serum levels of ALT and AST. Thrombocytopenia was present in 14 of 48 dogs (29.2%). According to Ciaramella et al. 2002 and 2005 [33], [34] thrombocytopenia with deficiency in platelet aggregation is directly related to the degree of clinical severity.

The difference in parasite load between the symptomatic and asymptomatic dogs has been evaluated by some researchers and asymptomatic dogs have lower loads than symptomatic ones. Sanchez et al. 2004 [35] demonstrated higher liver and spleen parasite loads in naturally infected symptomatic dogs from an endemic area of Venezuela. In Brazil, Giunchetti et al. 2008 [36] and Melo et al. 2009 [37] described similar results with the livers of dogs naturally infected with *L. infantum.* Moreover, in skin, a positive correlation among tissue parasite load, a chronic inflammatory reaction and symptomatic infection in dogs has been demonstrated by Giunchetti et al. 2006 [38], Xavier et al. 2006 [39] and Figueiredo et al. 2010 [40]. Considering this parasite load in skin and *L. longipalpis* infectivity (xenodiagnosis), it has been demonstrated that asymptomatic dogs are able to infect *L. longipalpis* as symptomatic dogs, but in lower proportions [29], [41]. In the present study, all infected dogs were classified as symptomatic. After parasitological analysis of the ear skin, the percentage of positive dogs was 54.16% and 56.25% using IHQ and XENO, respectively. Otherwise, 21 IHQ^+^/XENO^+^ dogs (43.75%) and 16 IHQ^−^/XENO^−^dogs (33.33%) represented the majority of cases where it means that there was a strong correlation between IHQ and XENO (r = 0.5373; p = 0.001). In contrast to the findings of Travi et al. 2001 [42] and Verçosa et al. 2008 [43], who used PCR-H and cytological skin imprints to detect the parasite, respectively, in our work we found a strong correlation between the parasite ear tissue load and the infectivity to *L. longipalpis*, which might provide a steadfast predictor of dogs infectivity to the vector.

Bazzocchi et al. 2005 [44] carried out flow cytometry assays using cross-reactive anti-human TLR2 antibodies to demonstrate TLR2 expression in canine granulocytes, monocytes and less markedly in lymphocytes from peripheral blood. In addition, Ishii et al. 2006 [45] evaluated dogs using semi-quantitative RT-PCR, and verified the presence of TLR2 mRNA in diverse canine cells and tissues including peripheral blood monocytes, lymph nodes, lung, liver, spleen, bladder, pancreas and skin. de Veer et al. [7] demonstrated that TLR2 is essential for the protective immune response against intracellular pathogens including *L. major* and *Toxoplasma gondii*.

Lipophosphoglycan (LPG), a major surface promastigote phosphoglycan purified from metacyclic promastigotes of *L. major,* promoted up-regulation of TLR2 expression in NK cells [8] and in IFN-γ-primed mouse macrophages. Flandin et al. 2006 [46] demonstrated that in relation to Galβ1 4Manα-PO_4_-containing phosphoglycans, TLR2 may act synergistically with TLR3 in recognizing *L. donovani* promastigotes. These authors also verified that when TLR2 or TLR3 are silenced using RNA interference, internalization of *L. donovani* promastigotes and the secretion of nitric oxide (NO) and TNF-α are reduced. Kavoosi et al. 2010 [47] demonstrated that NO production stimulated by *L. major* lipophosphoglycan was significantly decreased in macrophage cultures pretreated with anti-TLR2, and concluded that TLRs are important in *Leishmania* infection, inducing NO production via the TLR2 signaling pathway. Therefore, TLR2 recognizes a myriad of unrelated molecules [48], and their role in infection by *Leishmania* has been evaluated but is still controversial. However, Tuon et al. 2008, Kavoosi et al. 2010 and Guerra et al 2010 [10], [47], [49] have described the potential anti-parasite effector role of TLR2 in leishmaniasis experimental models. As far as we are aware, despite the importance of mammalian TLRs in leishmaniasis in general, there have been no studies concerning TLR2 in CVL.

The integrin CR3, a heterodimer of CD11b and CD18 and a known complement receptor for ingress of the *Leishmania* parasite into monocyte/macrophage lineage host cells, has diverse functions in immunity, adhesion and cell migration [50]–[53]. However, researchers hold differing views concerning the true role of CR3 in the establishment and progression of *Leishmania* infection in humans and dogs. de Almeida et al. 2003 [54], evaluating the expression of cell adhesion and co-stimulatory molecules on human monocytes after *in vitro* infection with *L. infantum* (MHOM/BR/90/Ba 307), verified by flow cytometry that CD11b expression was decreased. These authors considered that the inhibition of CD11b expression could be an escape mechanism. Interestingly, Marth et al. 1997 [55] verified that even in the absence of *Leishmania* infection the linking in CR3 promoted a reduction in IL-12 production, IL-12 having been key to the subsequent induction, magnitude and memory of the type I response. This immunological status could also limit NK-cell activation, which induces cytokines such as IFN-γ and TNF-α, which are responsible for the cellular immune response [8]. In CVL, it has been reported that CR3 could be important in the interaction among peripheral blood monocytes cells, monocyte-derived macrophages and peritoneal macrophages in the presence of opsonized promastigote forms of *Leishmania* [25], [56], [57]. In addition, according to Triantafilou and Triantafilou (2002) [11], CR3 cooperates with TLRs in association with other receptors such as CD14 and MHC II.

In the literature, there is a discussion considering an organ specific immunity response in murine experimental visceral leishmaniasis [58], human visceral leishmaniasis [59] and canine visceral leishmaniasis [30], [31]. Following this kind of stratagem, the evaluation of canine PBMC by flow cytometry in association to a parasitological skin appraisal, first site of contact with the *Leishmania*, could be relevant for investigation to a better understanding of CVL pathogenesis.

In this study we have investigated CR3 and TLR2 expression in relation to the skin parasite load (IHQ) and XENO. A direct relation between TLR2 and CR3 (r = 0.6937) was identified, suggesting some kind of activation between both receptors. Following these analyses, a comparison inside groups of dogs with IHQ and XENO with double positive or negative results was carried out. Symptomatic dogs with higher expression of CD11b (MFI) inside CD14 monocytes cells was represented by dogs without parasite ear tissue load (IHQ negative) that were unable to infect phlebotomines (XENO negative) (IHQ^-^/XENO^-^). On the other side, symptomatic dogs with lower expression of MFI of CD11b inside CD14 monocytes cells was represented by dogs with parasite ear tissue load (IHQ positive) and able to infect phlebotomines (XENO positive) (IHQ^+^/XENO^+^) (p = 0.0032). The same results were obtained for MFI of MHCII inside CD14 monocytes cells (p = 0.0054). In addition, considering the population frequency of CD11b^+^TLR2^+^ and CD11b^+^MHCII^+^, inside CD14 monocytes cells, higher values for dogs with IHQ^−^/XENO^−^ were demonstrated than for dogs with IHQ^+^/XENO^+^ (p = 0.01; p = 0.0048, respectively). Other studies in CVL have tried to show some correlations between distinct immune response and parasite tissue loads. Lage et al. 2007, in Brazil [60] in a study with 30 naturally infected dogs with *L. chagasi* found higher parasite load in spleens (eight dogs) in concordance to a significant increasing of IL-10 levels (p = 0.011), when compared with others with lower parasite spleen tissue load. These authors suggested a balanced production of cytokine of Th1 and Th2. In the another side, Strauss-Ayali et al. 2007 [61], in Israel, working with six experimentally and ten naturally infected dogs with *L. infantum* did not found a strict correlation between the spleen parasite tissue load and Th2 immune response. In fact, although the parasite load was higher in the polysymptomatic dogs than others the authors did not find statistical correlation between the parasite load and expression levels of any cytokines, transcriptions factors and chemokines (IFN-γ, TNF-α, IL-4, IL-5, IL-10, TGF-β, IP-10, RANTES, MIP-1α, MCP-1 and β-actin).

The induction of nitric oxide (NO) is one of the major effector mechanisms leading the *Leishmania* elimination by activated phagocytes [62]. Zafra et al. 2008 [63], in Spain, working with 33 naturally infected dogs with *L. chagasi* made a direct correlation between higher NO expression in tissues as skin, livers and popliteal lymph nodes and lower numbers of intracellular amastigotes forms of *Leishmania* by immunohistochemistry. In our research, we found that IHQ^−^/XENO^−^ dogs showed higher values than IHQ^+^/XENO^+^ dogs.

CR3 (CD11b) is an inflammatory protein that is very important during cellular extravasion (diapedesis) and higher expression of this molecule by monocytes infers the presence of an inflammatory reaction or effector cells (macrophages) in tissues or target organs. TLR2 in a transmembrane receptor expressed in the cell surface of immune cells, being activated by *Leishmania* LPG. This interaction leads to the production of inflammatory mediators as TNF-α, IL-12 and IFN-γ where TLR2 play a potential anti-parasite effector role [7], [8], [10]. However, in according to Chandra and Naik 2008 [64] TLR2 could be down-regulated by *Leishmania* (complex *L. donovani*) with an increasing IL-10 production by monocytes-macrophages. Symptomatic dogs with parasitological negative results by immunohistochemistry and xenodiagnosis analysis, defined as IHQ^−^/XENO^−^ group, showed higher expression levels of CD11b and MHC II by peripherical blood monocytes cells. These results could suggest that dogs IHQ^−^/XENO^−^ are more resistant than IHQ^+^/XENO^+^ dogs, because CD11b/MHCII monocytes receptors might play an effector role anti-parasite. However, we must consider the possibility of IHQ^−^/XENO^−^ dogs are not in protection against canine visceral leishmaniasis since all symptomatic animals had positive parasitological exams (amplification of kDNA of *Leishmania*) from bone marrow aspirates. These data, together with the TLR2 and NO assays results (CD11b^+^TLR2^+^ and NO with higher values for dogs with IHQ^−^/XENO^−^ than dogs with IHQ^+^/XENO^+^), led to the conclusion that IHQ^−^/XENO^−^ dogs are more resistant or could modulate the cellular immune response essential for *Leishmania* tissue clearance.
